# Short-Chain Fatty Acids Augment Differentiation and Function of Human Induced Regulatory T Cells

**DOI:** 10.3390/ijms23105740

**Published:** 2022-05-20

**Authors:** Mingjing Hu, Bilal Alashkar Alhamwe, Brigitte Santner-Nanan, Sarah Miethe, Hani Harb, Harald Renz, Daniel P. Potaczek, Ralph K. Nanan

**Affiliations:** 1 Charles Perkins Centre Nepean, Sydney Medical School Nepean, The University of Sydney, Sydney, NSW 2747, Australia; mihu9213@uni.sydney.edu.au (M.H.); brigitte.nanan@sydney.edu.au (B.S.-N.); 2 International Inflammation (in-VIVO) Network, Worldwide Universities Network (WUN), West New York, NJ 10001, USA; bilal.alashkaralhamwe@staff.uni-marburg.de (B.A.A.); hani.harb@ukdd.de (H.H.); renzh@med.uni-marburg.de (H.R.); daniel.potaczek@staff.uni-marburg.de (D.P.P.); 3 Discipline of Obstetrics, Gynaecology and Neonatology, Sydney Medical School Nepean, The University of Sydney, Sydney, NSW 2747, Australia; 4 Nepean Hospital, Derby Street, Kingswood, NSW 2747, Australia; 5 Institute for Tumor Immunology, Clinic for Hematology, Immunology, and Oncology, Philipps University Marburg, 35043 Marburg, Germany; 6 Institute of Laboratory Medicine, Philipps University of Marburg-Medical Faculty, Member of the German Center for Lung Research (DZL), and the Universities of Giessen and Marburg Lung Center (UGMLC), 35043 Marburg, Germany; sarah.miethe@staff.uni-marburg.de; 7 College of Pharmacy, International University for Science and Technology (IUST), Daraa 15, Syria; 8 Translational Inflammation Research Division & Core Facility for Single Cell Multiomics, Philipps University of Marburg-Medical Faculty, Member of the German Center for Lung Research (DZL) and the Universities of Giessen and Marburg Lung Center (UGMLC), 35043 Marburg, Germany; 9 Institute of Medical Microbiology and Virology, Medical Faculty, Technische Universität Dresden, 01307 Dresden, Germany

**Keywords:** short-chain fatty acids, butyrate, propionate, regulatory T cells, histone acetylation

## Abstract

Regulatory T cells (Tregs) control immune system activity and inhibit inflammation. While, in mice, short-chain fatty acids (SCFAs) are known to be essential regulators of naturally occurring and in vitro induced Tregs (iTregs), data on their contribution to the development of human iTregs are sparse, with no reports of the successful SCFAs-augmented in vitro generation of fully functional human iTregs. Likewise, markers undoubtedly defining human iTregs are missing. Here, we aimed to generate fully functional human iTregs in vitro using protocols involving SCFAs and to characterize the underlying mechanism. Our target was to identify the potential phenotypic markers best characterizing human iTregs. Naïve non-Treg CD4^+^ cells were isolated from the peripheral blood of 13 healthy adults and cord blood of 12 healthy term newborns. Cells were subjected to differentiation toward iTregs using a transforming growth factor β (TGF-β)-based protocol, with or without SCFAs (acetate, butyrate, or propionate). Thereafter, they were subjected to flow cytometric phenotyping or a suppression assay. During differentiation, cells were collected for chromatin-immunoprecipitation (ChIP)-based analysis of histone acetylation. The enrichment of the TGF-β-based protocol with butyrate or propionate potentiated the in vitro differentiation of human naïve CD4^+^ non-Tregs towards iTregs and augmented the suppressive capacity of the latter. These seemed to be at least partly underlain by the effects of SCFAs on the histone acetylation levels in differentiating cells. GITR, ICOS, CD39, PD-1, and PD-L1 were proven to be potential markers of human iTregs. Our results might boost the further development of Treg-based therapies against autoimmune, allergic and other chronic inflammatory disorders.

## 1. Introduction

Regulatory T cells (Tregs) control immune system activity and inhibit the inflammatory process. In subjects suffering from autoimmune disorders, Tregs are compromised [1,2,3]. Therapeutic strategies aiming to boost Treg cell activity by either increasing the number or enhancing the function of already existing Tregs, or reinfusing them after prior ex vivo expansion, have reached the phase of clinical trials. Compared to non-specific immunosuppressive drugs, Tregs are safer, more effective, and have fewer side effects [1,2,3]. The contribution of reduced Treg function to disease development has also been demonstrated for allergic disorders, such as atopic asthma, allergic rhinitis, food allergies, atopic dermatitis, allergic conjunctivitis, and others [4,5,6,7,8,9,10,11]. Additionally, in this case, various types of therapeutic interventions involving Tregs have been proposed [12,13,14,15,16,17].

Short-chain fatty acids (SCFAs), the major end products of bacterial fermentation in the human colon, play an important role in protection against several chronic inflammatory or autoimmune diseases, such as asthma, allergies, inflammatory bowel disease, and type 1 diabetes, mostly through their putatively significant role in Treg induction [1,18,19,20,21,22,23]. Indeed, the substantial contribution of SCFAs to the differentiation of mouse Tregs extrathymically both in vivo or in vitro, so-called induced Tregs (iTregs), has been shown [24,25,26,27]. Data on the effects of SCFAs on human Tregs are sparse and the successful in vitro development of fully functional human iTregs using a SCFAs-augmented protocol has not been reported yet [18,26,27,28,29].

Forkhead box P3 (Foxp3) is currently considered to be the most specific marker for naturally occurring Tregs, especially those generated in the thymus. However, growing evidence from humans suggests that this issue is more complex. For instance, there is also a CD4^+^Foxp3^+^ subpopulation in human peripheral blood (PB) possessing no suppressive capacity [30]. Some human T cells can transiently up-regulate Foxp3 expression upon in vitro stimulation without acquiring Treg-cell suppressive properties [31,32]. In addition to Foxp3, other markers, including glucocorticoid-induced TNF-related protein (GITR) [33,34,35], CD39 [36,37,38], inducible T-cell costimulator (ICOS, CD278) [39,40], programmed cell death protein 1 (PD-1, CD279), programmed cell death ligand 1 (PD-L1, CD274) [41,42], interleukin-7 receptor subunit alpha (CD127) [43], and human leukocyte antigen–antigen D-related (HLA-DR) [44], have been investigated, but there is still no definitive marker undoubtedly defining human iTregs.

Establishing further human iTreg generation strategies and a better understanding of their biology will boost the development of Treg-based therapeutic approaches. Hence, we aimed to successfully generate fully functional human iTregs in vitro using protocols involving SCFAs and to characterize the mechanism underlying this differentiation. In parallel, we wanted to identify the potential phenotypic markers best characterizing human iTregs.

## 2. Results

### 2.1. Differential Activation Patterns in the Generation of Human Tregs In Vitro via SCFAs

To compare the iTreg cell induction patterns between cord blood (CB) mononuclear cells (CBMCs) and adult peripheral mononuclear cells (PBMCs), FACS-sorted naïve non-Treg cells (CD4^+^CD45RO^−^CD25^−^CD127^hi^) were stimulated with anti-CD3/anti-CD28 in the presence of interleukin-2 (IL-2) and transforming growth factor β (TGF-β), with or without SCFAs, for 5 days (Supplementary Figure S1).

**Figure 1:**
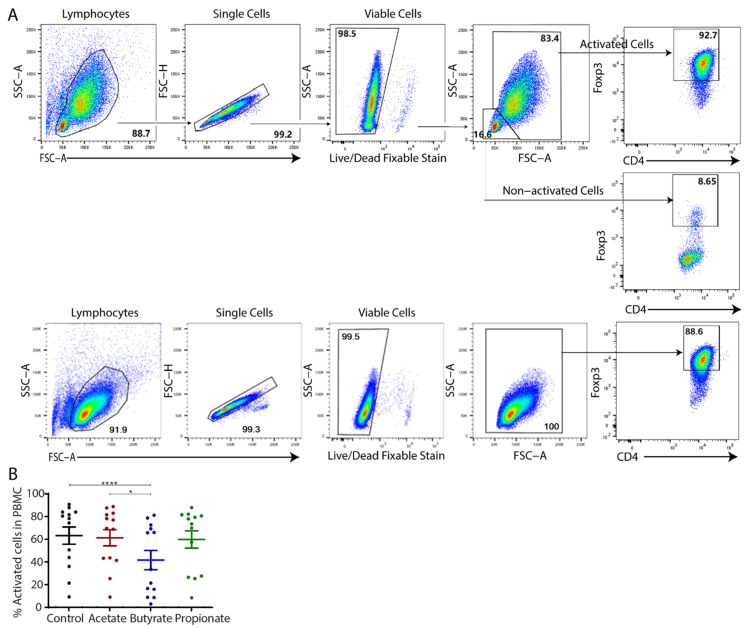
Differential activation patterns between adult and cord blood in the generation of human in vitro induced regulatory T cells (iTregs) in the absence or presence of short-chain fatty acids (SCFAs). (**A**) Flow cytometric gating strategy for activated cells gated on viable cells after Treg induction from adult PBMC (upper panel) and CBMC (lower panel) naïve CD4^+^ cells. (**B**) Scattered dot plot with the mean and standard error of the mean (SEM) showing the percentage of activated Fop3^+^ cells after induction from adult (left) naïve CD4^+^ cells under various conditions, summarizing thirteen independent experiments. Friedman test with Dunn’s multiple comparisons (post hoc) was used for statistical analysis. * *p* < 0.05, **** *p* < 0.0001.

The induction patterns differed between CBMCs and adult PBMCs in terms of their activated cell proportions (Figure 1). Naïve non-Tregs from adult PBMCs were unable to fully differentiate into Foxp3^+^ iTreg cells (Figure 1A). After gating on viable cells induced from adult PBMCs, we noted that, apart from the commonly known “activated” iTreg cells, there was a significant population of smaller Foxp3-negative cells, representing “non-activated” cells. The percentages of activated cells varied significantly across the study population and under different culture conditions (Figure 1B). In particular, iTregs exposed to butyrate had the smallest proportion of activated cells. In contrast, all of the naïve non-Tregs from CBMCs differentiated fully into activated Treg cells, independent of SCFAs exposure (Figure 1A).

### 2.2. Butyrate and Propionate, but Not Acetate, Potentiate the Expression of Phenotypic Markers of Human TGF-β-Induced Tregs In Vitro

**Figure 2:**
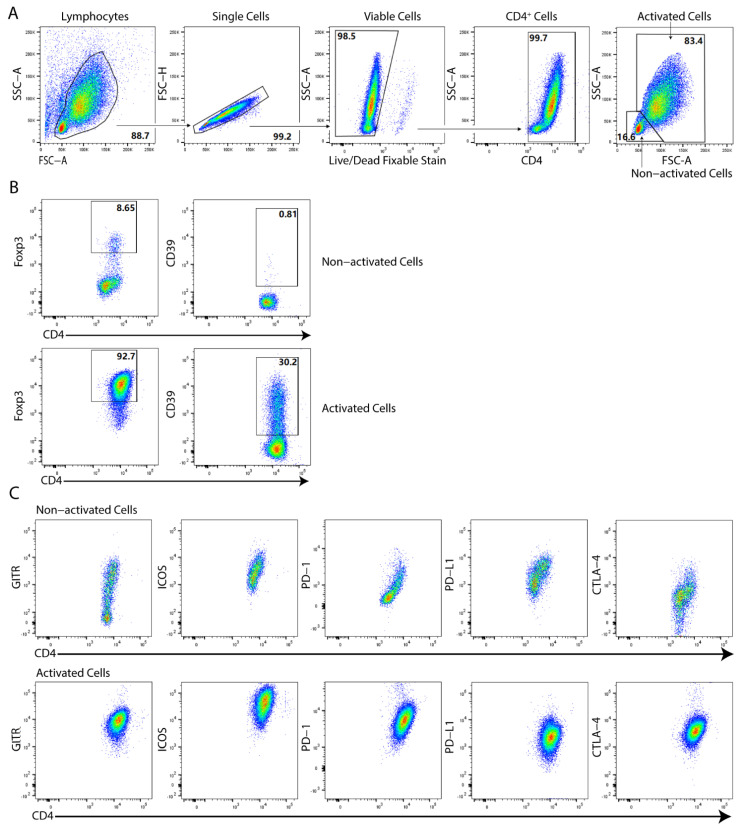
Flow cytometric gating strategy for the phenotypic markers of human in vitro induced regulatory T cells (iTregs) generated from adult naïve CD4^+^ cells. (**A**) Flow cytometric gating strategy for lymphocytes, single cells, viable cells, CD4^+^ cells, and non-activated and activated iTregs generated from adult naïve CD4^+^ cells. (**B**) Representative dot plots showing the percentages of Foxp3 and CD39 gated on either non-activated (left panels) or activated (right panels) iTreg cells. (**C**) Representative dot plots showing the GITR, ICOS, PD-1, PD-L1, and CTLA-4 expression patterns on either non-activated or activated iTreg cells generated from adult naïve CD4^+^ cells with propionate. Data summarize thirteen independent experiments.

**Figure 3:**
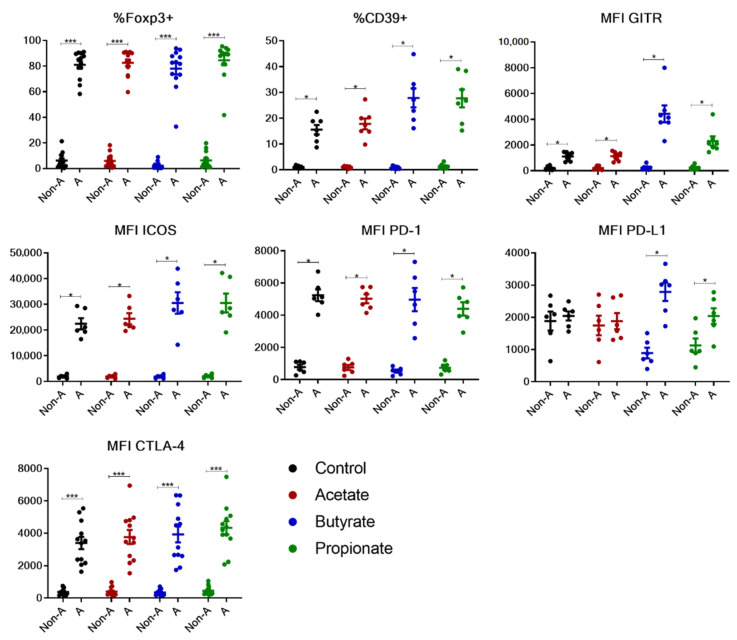
Enhanced expression of phenotypic markers in activated cells generated from adult naïve CD4^+^ cells. Scattered dot plot with mean + SEM showing the percentage of Foxp3^+^ and CD39^+^ cells, and mean fluorescence intensity (MFI) of GITR, ICOS, PD-1, PD-L1, and CTLA-4 gated on non-activated (Non-A) and activated (A) cells generated from adult naïve CD4^+^ cells under various conditions. All cells were cultured in the presence of transforming growth factor β and interleukin-2, and additionally with control, acetate, butyrate, or propionate. Data summarize thirteen independent experiments. Wilcoxon matched-pairs rank test was used for statistical analysis. * *p* < 0.05, *** *p* < 0.001.

There were obvious differences between non-activated and activated cells generated from adult PBMCs in terms of the expression levels of various phenotypic markers, as exemplified in Figure 2. Compared to non-activated cells, the activated iTregs generated under various conditions (control, acetate, butyrate, or propionate) had higher expression levels of the phenotypic markers tested, except for PD-L1 in the control and acetate-treated iTreg cells (Figure 3). A similar trend was observed for the phenotypic markers within the Foxp3^+^ cells between the activated and non-activated cells (Supplementary Figure S2).

**Figure 4:**
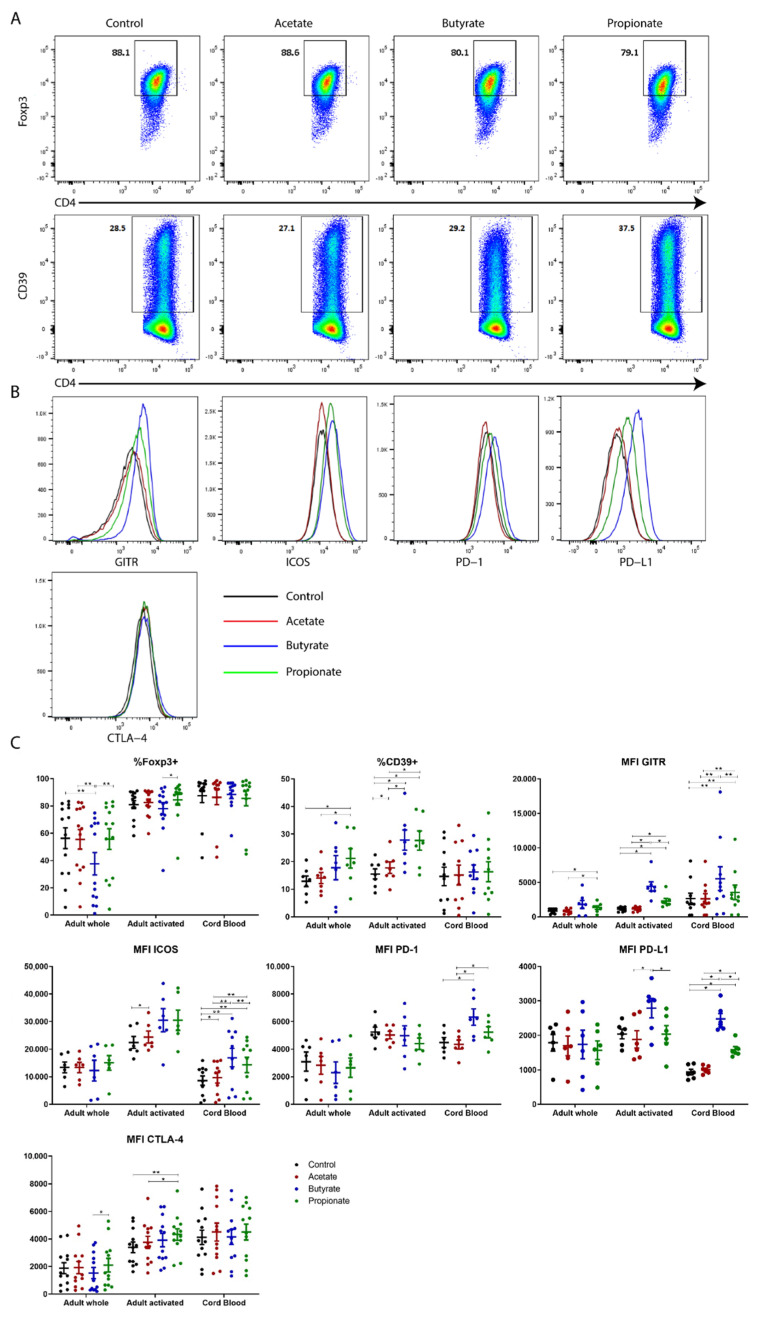
Short-chain fatty acids (SCFAs) potentiate the generation of human transforming growth factor β (TGF-β)-induced Tregs in vitro. (**A**) Representative dot plots gated on live CD4^+^CD3^+^ T cells showing the percentage of Foxp3^+^ (upper panel) and CD39^+^ (lower panel) cells induced after culturing under various conditions: control (interleukin-2 (IL-2) + TGF-β), acetate (IL-2 + TGF-β + acetate), butyrate (IL-2 + TGF-β + butyrate), and propionate (IL-2 + TGF-β + propionate). (**B**) GITR, ICOS, PD-1, PD-L1, and CTLA-4 expression of live CD4^+^CD3^+^ T cells cultured under various conditions (see the legend to Panel A), as shown by representative histograms. (**C**) Scattered dot plots showing the mean and standard error of the mean (SEM) summarizing thirteen independent experiments for adults and twelve independent experiments for cord blood. Friedman test with Dunn’s multiple comparisons (post hoc) was used for statistical analysis. * *p* < 0.05, ** *p* < 0.01.

The expression levels of human iTreg phenotypic markers in cells differentiated under various conditions are presented in Figure 4. Due to the significantly higher percentage of non-activated cells, butyrate-treated iTregs from adult PBMCs had the lowest Foxp3 expression compared to the other cells treated with other SCFAs (Figure 4C). The expression of Foxp3 was similar among the groups when analyzing only the activated cells, as well as iTreg cells generated from CBMCs (Figure 4C). As for CD39, GITR, ICOS, and PD-L1 expression, the trend was the same for both activated iTregs from adult PBMCs and iTregs from CBMCs (Figure 4C). Butyrate-treated iTregs had the highest expression for these four markers, followed by propionate-treated iTregs, though not always at statistically significant levels. The expression levels of these markers of iTregs treated with acetate were either the same or only slightly higher compared to the control (Figure 4C). Butyrate-/propionate-treated iTregs generated from CBMCs had significantly higher expression of PD-1 than acetate-treated or control iTreg cells (Figure 4C). However, this trend was not seen in iTregs from adult PBMCs. The CTLA-4 expression levels were, in turn, higher in the propionate-treated activated iTregs from adult PBMCs than in the control or acetate-treated cells, although those differences were biologically small (Figure 4C). When comparing the phenotypic markers between adult activated iTregs and iTregs from CBMCs, the expression levels of most phenotypic markers were similar (Supplementary Figure S3).

Cumulatively, these results suggest that (a) Foxp3 is mainly an activation marker for human iTregs; (b) GITR, ICOS, CD39, PD-1, PD-L1, and CTLA-4 are potential markers for human iTregs; and (c) butyrate and propionate, but not acetate, potentiate the expression of phenotypic markers of human TGF-β-induced Treg cells in vitro.

### 2.3. Butyrate and Propionate, but Not Acetate, Enhance the Suppressive Capacity of Human TGF-β-Induced Tregs In Vitro

To test the suppressive capacity of iTreg cells treated with different SCFAs, these iTreg cells were co-cultured with allogeneic CFSE-labeled CD4^+^CD25^−^ responder cells (freshly bead-isolated) under various ratios in vitro. Then, on day 3, they were analyzed for the differences in the proliferation of responder cells.

**Figure 5:**
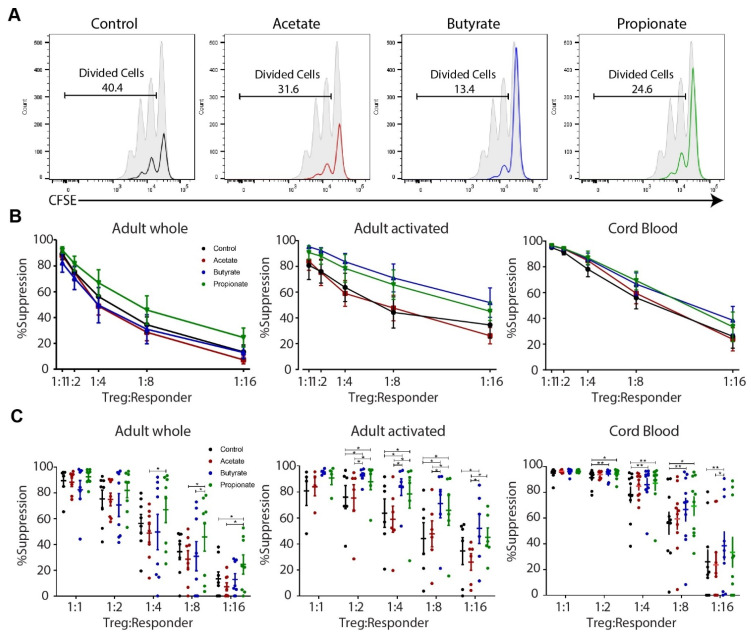
Short-chain fatty acids (SCFAs) enhance the suppressive capacity of in vitro induced regulatory T cells (iTregs). (**A**) Representative histograms showing the proliferation of responder cells co-cultured with iTregs differentiated under various conditions (see legends to Figure 4); data shown correspond to a suppressor/responder ratio of 1:8. (**B**,**C**) Percentage of suppression obtained at varying suppressor/responder ratios using the whole population (left panels; eight independent experiments) or only the population of activated (bead-selected CD25^high^; middle panels; six independent experiments) iTregs deriving from adult naïve CD4^+^ cells, and iTreg cells deriving from cord blood naïve CD4^+^ cells (right panels; ten independent experiments) differentiated under various conditions (see legends to Figure 4). The data are given as means with the standard error of the mean (SEM). Friedman test with Dunn’s multiple comparisons (post hoc) was used for statistical analysis. * *p* < 0.05, ** *p* < 0.01.

Firstly, considering the significantly different activation statuses reported in the previous section, we wanted to test the suppressive capacity of the activated iTreg cells generated from adult naïve CD4^+^ cells. To isolate activated (purified CD25^high^) cells for the in vitro suppression assay, the whole population of cultured cells incubated under various conditions (control, acetate, butyrate, or propionate) was subjected to CD25-based positive selection. Butyrate or propionate-treated iTreg cells reduced the in vitro proliferation of allogeneic CD4^+^CD25^−^responder cells compared to the control iTreg cells (Figure 5). This was statistically significant and consistent at all suppressor/responder cell ratios, i.e., from 1:2 to 1:16. On the contrary, the addition of acetate failed to achieve a similar effect (Figure 5).

Finally, we tested the suppressive capacity of iTreg cells generated from CB naïve CD4^+^ cells. The results mimicked the trend observed in activated iTregs generated from adult naïve CD4^+^ cells (Figure 5). The addition of butyrate or propionate increased the suppressive capacity of iTregs at all suppressor/responder cell ratios, ranging from 1:1 to 1:16. Significant differences were found at ratios ranging from 1:2 to 1:16 for butyrate-treated iTregs (Figure 5). As for propionate-treated iTregs, there was significant suppression at ratios from 1:2 to 1:8. Like the activated iTregs generated from adult naïve CD4^+^ cells, the addition of acetate was unable to increase the suppressive capacity of iTreg cells generated from CB naïve CD4^+^ cells (Figure 5).

Due to the high suppressive capacity of these iTregs (>80%), significant differences were masked in the higher suppressor/responder cell ratios among different conditions. In contrast to lower proportions of iTregs, the differences became more obvious and statistically significant.

These results demonstrate that the addition of butyrate and propionate, but not acetate, augments the suppressive capacity of in vitro induced human Treg cells.

### 2.4. Butyrate and Propionate Affect Histone Acetylation Levels at Important Regulatory Regions of Genes Encoding Molecules Crucial for Treg Function

**Figure 6:**
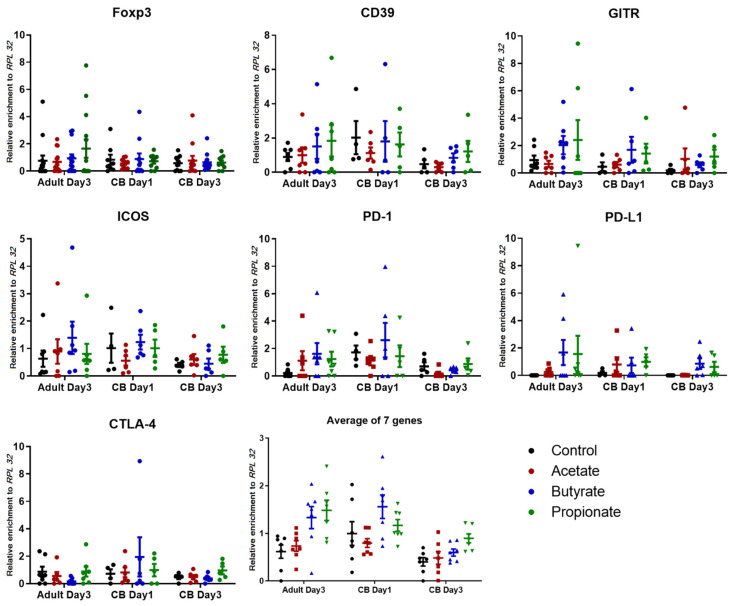
Histone H4 pan-acetylation status at the transcription-controlling regions of regulatory T cell (Treg) genes. Histone 4 acetylation levels at seven Treg loci given as the relative enrichment to ribosomal protein L32 (*RPL32*) in human adult (n = 7; peripheral blood) or newborn (n = 6; cord blood, CB) naïve CD4^+^ non-Treg cells differentiated toward iTreg phenotype in the presence or absence of short-chain fatty acids (SCFAs). Adult-derived cells were harvested on day 3, and CB-originating cells on days 1 and 3 (see Supplementary Figure S1). H4 acetylation was analyzed by a chromatin immunoprecipitation–quantitative polymerase chain reaction (ChIP-qPCR) assay. The data are expressed as means with the standard error of the mean (SEM). There was no statistical significance using the Friedman test with Dunn’s multiple comparisons (post hoc).

Searching to find a potential mechanism of how SCFAs augment iTreg differentiation and functioning, naïve CD4^+^ non-Treg cells derived from CBMCs or PBMCs, cultured toward iTregs in the presence or absence of SCFAs and harvested on days 1 and 3 or day 3, respectively (Supplementary Figure S1), were studied for the histone H4 acetylation status at important regulatory regions of several iTreg-specific loci, such as genes encoding CD39, CTLA-4, PD-1, GITR, ICOS, PD-L1, and Foxp3 (Supplementary Table S1). Considering that SCFAs treatment inhibits global histone deacetylation, the chromatin immunoprecipitation–quantitative polymerase chain reaction (ChIP-qPCR) assay output values within each sample were normalized, not only to the input and isotype control, but also to the positive control gene. The results of the ChIP-qPCR investigations are given in (Figure 6) and Supplementary Table S2.

Overall, the average (Figure 6) and cumulative (Supplementary Table S2) histone H4 pan-acetylation (K5ac, K8ac, K12ac, and K16ac) at the Treg loci was the highest in cells treated with butyrate or propionate, whereas the acetylation levels observed in cells exposed to acetate were in most cases not much different from those in untreated cells. From the single-gene perspective, the strongest effects on H4 acetylation were seen in PBMC-derived cells harvested on day 3 for Foxp3 (propionate), CD39 (butyrate and propionate), GITR (butyrate and propionate), ICOS (butyrate), PD-1 (acetate, butyrate and propionate), and PD-L1 (butyrate and propionate), in CBMC-originating cells collected on day 1 for GITR (butyrate and propionate), PD1 (butyrate), PD-L1 (acetate, butyrate, and propionate), and CTLA-4 (butyrate), and in CB cells harvested on day 3 for CD39 (butyrate and propionate), GITR (acetate and propionate), ICOS (propionate), PD-L1 (butyrate and propionate), and CTLA-4 (propionate) (Figure 6).

Collectively, these results might indicate that, in naïve CD4^+^ non-Treg PBMC- or CBMC-derived cells differentiating toward iTregs, butyrate and propionate, and, to a lesser extent, enhance the histone H4 acetylation status at several Treg-related loci.

## 3. Discussion

In mice, SCFAs have been well established as essential immune regulators of naturally occurring and in vitro induced Tregs. However, data on the contribution of SCFAs to the development of human Tregs, especially iTregs, are sparse, with no reports of the successful in vitro generation of human iTregs possessing full suppressive abilities using a SCFA-augmented protocol [18,26,27,28,29,45]. Generally, it remains inconclusive whether human Treg cells developed in vitro using SCFAs or other substances [45,46,47,48,49,50] are able to obtain suppressive capacities comparable to those of ex vivo-cultured or in vivo-developed natural Tregs and if they can remain stable for longer periods of time [51,52,53]. Finally, in the only study involving SCFAs reported so far, protocols applying butyrate or propionate failed to generate functional human iTregs possessing reasonable suppressive abilities [45].

In the present study, we tested a human iTreg differentiation protocol applying the SCFAs acetate, butyrate, or propionate, in addition to the classically used IL-2 and TGF-β [45,46,47,48,49,50]. We found that the addition of butyrate or propionate, but not acetate, significantly enhanced the suppressive capacity of human in vitro generated iTregs. This observation was most obvious in iTregs of CB origin and in activated iTregs generated from adult naïve CD4^+^ T cells. The lack of a need for the additional activation of CB-deriving iTregs might result from the much higher percentage of naïve, “inducible” cells present in CB. Furthermore, the differences in the induction patterns observed between different origins (CBMCs or adult PBMCs) might explain the controversies regarding the suppressive effectiveness of human iTregs [51,52,53], as well as the concealment of the effects of SCFAs on adult cells differentiated towards iTregs [45].

In our study, naïve non-Treg cells isolated from adult CD4^+^ cells were unable to fully differentiate into Tregs, and there was a significant non-activated subpopulation with a smaller cell size. Among all of the culture conditions, butyrate-treated iTregs had the most non-activated cells, around 40% of the whole cultured cells. We speculated that this population was nonresponsive and did not share iTreg characteristics. Indeed, these cells had the minimal expression of all the phenotypic markers. We then compared the suppressive capacity between activated iTreg cells and their pair-matched whole cultured cells generated from adult naive CD4^+^ cells. As expected, the activated iTregs demonstrated a higher percentage of suppression compared to their non-activated counterparts. In butyrate-treated iTregs, these differences were much greater when the large proportion of non-activated cells was taken into account. Interestingly, this differential induction pattern was not present in the iTregs generated from CB naïve CD4^+^ cells. All naïve non-Tregs sorted from CB naïve CD4^+^ cells showed full differentiation into iTregs. These findings are further supported by the fact that the histone acetylation changes most probably at least in part resulting from SCFAs treatment were observed in CB cells earlier than in adult cells. This reasoning is limited by the lack of early time point epigenetic data for the latter. Nevertheless, when analyzing iTregs generated from CB naïve CD4^+^ cells and the activated, rather than the whole, iTregs generated from adult PB naïve CD4^+^ cells, we were able to reproduce the results of the relevant animal studies.

Considering the lack of methodological differences between our experiments conducted using cells deriving from CB and PB, the question arises as to whether CD45RO^−^ T cells are truly “naïve” in adults, especially compared to CB. Human naïve T cells express CD45RA, but lack the expression of CD45RO, and the expression of at least one additional naïve T cell marker, such as CCR7, CD62L, or CD27, is necessary to distinguish naïve from terminally differentiated T cells [54,55]. The neonatal immune system is largely dominated by truly naïve T cells [54]. Although the size and scope of the naïve CD4^+^ T cell pool tends to be maintained by both thymic output and peripheral homeostatic proliferation throughout adulthood [56,57], there is a progressive reduction of the thymus output with a resulting decline in the naïve T cell pool and expansion of the oligoclonal T cell populations [58]. In addition, there are functional alterations in the responsiveness of naïve T cells in elderly individuals, which may be the result of a shift from truly naïve to more mature naïve-like T cells [55,56,57,58,59]. Indeed, T cell receptor sequencing studies have shown that clonal expansion also occurs in human naïve T cells, and most of these clonally expanded cells still retain a naïve phenotype with the expression of CD45RA, CD28, CD27, and CCR7 [60]. In addition, telomere attrition, DNA damage, as well as changes in the microRNA spectrum occur in naïve T cells when aging, which partially account for defective T cell responses [61,62]. However, conclusive data on the transcriptional and epigenetic profiling exploring the influence of aging on naïve T cells in humans are still missing.

Our epigenetic investigations demonstrated the possible mechanism underlying the SCFA-mediated augmentation of the differentiation towards human iTreg cells. Butyrate, propionate, and, to a lesser extent, acetate most probably at least partly enhanced histone acetylation at the important regulatory regions of several Treg-associated loci, thus increasing the accessibility of these genes to the transcriptional machinery [63,64,65]. Our observations seem to be in line with the contribution of changes in histone acetylation patterns to the SCFA-boosted Treg differentiation reported previously in mice [26,27,28]. However, the possibility that the effects of SCFAs on in vitro human iTreg generation are also mediated by other mechanisms, such as energy metabolism [66,67], cannot be excluded. Finally, the differentiation of human iTregs corresponding to the expression of crucial related molecules, such as CD39, CTLA-4, PD-1, GITR, ICOS, PD-L1, and Foxp3, is subject to complex regulation involving not only environmental (e.g., SCFAs), but also genetic, factors, and their mutual interaction. Thus, even though it is beyond the scope of the present study, it is worth mentioning that certain relevant genetic factors have been identified. For example, a clear regulatory effect has been demonstrated in the case of CD39 expression driven by a variant in the ectonucleoside triphosphate diphosphohydrolase 1 gene (*ENTPD1*) encoding this molecule [68,69,70]. The huge complexity of gene expression regulation has not only biological, but also experimental, consequences in the form of much higher heterogeneity in humans compared to the rather homogenous results generated in in-bred animal models with highly controlled environmental conditions and a uniform genetic background. Thus, considering that histone acetylation is highly important, but also susceptible to experimental fluctuations in humans, this facultative part of our results needs to be interpreted with caution.

Along with the previous reports [30,31,32], the expression patterns we observed for Foxp3 in human in vitro-induced iTregs suggested it to be only an iTreg activation marker. We also demonstrated that the iTreg patterns of GITR, ICOS, CD39, PD-1, and PD-L1 expression corresponded to the actual suppressive abilities of those cells, as demonstrated by our functional assays. Thus, those five molecules might represent potential candidate markers for human in vitro induced Treg cells.

In conclusion, the successful SCFA-augmented in vitro generation of human iTreg cells and its mechanistic characterization reported here adds to the current knowledge on iTreg biology, creates a potentially useful tool for further research, and might boost the further development of Treg- and, considering the origin of natural SCFAs in human organisms, personalized nutrition-based therapeutic strategies against autoimmune, allergic, and other chronic inflammatory disorders.

## 4. Materials and Methods

### 4.1. Participants

This research was reviewed and approved by the Human Research Ethics Committee (HREC) of Nepean Blue Mountains Local Health District according to the Declaration of Helsinki. All participants were recruited on a volunteer basis and signed a written informed consent prior to donating blood.

To determine the effect of SCFAs on Tregs differentiation from naïve non-Treg cells, CBMCs were also used in the study in addition to adult PBMCs. Shielded through the placenta, CB is more genuinely “naïve” as it has limited environmental antigen exposure.

In total, 13 healthy adult volunteers (average age of 29.15 ± 1.92 years old with an average of 48.09% naïve CD4 T cells in PBMCs) and 12 healthy term pregnant women were recruited. Individuals with any history of autoimmune diseases or allergies were excluded. Adult blood samples were drawn from the antecubital vein. CB samples were collected via the sterile aspiration of the umbilical vein from the delivered placenta immediately after an elective caesarean section (CS). All 12 CS were due to repeated CS, malpresentation or other non-medical issues. Women with gestational diabetes, chorioamnionitis, placenta previa, chronic villitis, or twin pregnancies were excluded. The overall design of the study is presented in Supplementary Figure S1.

### 4.2. Mononuclear Cell Isolation

All blood samples were processed within 2 h of collection. Human MCs were isolated from blood using Ficoll-Paque PLUS density gradient medium (GE Healthcare Life Sciences, Pittsburgh, PA, USA). Blood collected in heparinized vacuum tubes was diluted in a 1:1 ratio with sterile phosphate-buffered saline (PBS; pH 7.4; Sigma–Aldrich, St. Louis, MO, USA). This mixture was carefully layered over Ficoll-Paque PLUS medium at a 2:1 ratio before centrifugation at 1700 rpm for 30 min, without brake. The layer containing MCs was carefully aspirated and washed twice in PBS with 0.5% fetal calf serum (FCS; Thermo Fisher Scientific, Waltham, MA, USA) at 1300 rpm for 8 min. MCs were then re-suspended in PBS containing 0.5% FCS. After trypan blue-based counting, MCs were subjected to cell sorting.

### 4.3. Fluorescence-Activated Cell Sorting (FACS)

Freshly isolated cells were surface stained with the appropriate fluorochrome-conjugated antibody, including anti-CD127 (FITC, 560549, Becton Dickinson, San Jose, CA, USA), anti-CD45RO (PerCp-eFluor710, 130-097-590, Miltenyi Biotec, Bergisch Gladbach, Germany), anti-CD4 (V500, 562970, Becton Dickinson), and anti-CD25 (APC, 340939, Becton Dickinson). The cells were sorted on a BD FACSAria^TM^ (Becton Dickinson) cell sorter. For Treg induction, the pre-enriched CD4^+^ T cells (PBMCs were labeled with a Miltenyi CD4^+^ T cell Isolation Kit #130-091-155 and separated on a Cell Separation Magnet) were further sorted for CD4^+^CD45RO^−^CD25^−^CD127^hi^ with a purity of ≥99%.

### 4.4. In Vitro Cultures

FACS-sorted naïve non-Treg cells of both adult and CB origins were cultured in cell culture medium (RPMI-1640 (Thermo Fisher Scientific) containing L-Glutamine (2mM, Sigma-Aldrich), penicillin (100 U/mL, Sigma-Aldrich), streptomycin (100μg/mL, Sigma-Aldrich) plus 1% HEPES (Thermo Fisher Scientific), and 10% FCS in 48-well plates, with immobilized anti-CD3 (OKT3, 10µg/mL, coated overnight in PBS at 4 °C, 317304, Biolegend, San Diego, CA, USA) and mobilized anti-CD28 (2 µg/mL, 302914, BioLegend. Cells were cultured at a concentration of 3.5–5 × 10^5^/48 wells at 37 °C for 5 days under standard culture conditions (37 °C, pH 7.4, 5% CO_2_, 85–95% humidity). Recombinant human IL-2 (50 U/mL, 130-097-745, Miltenyi Biotec), TGF-β (5 ng/mL, 100-21, Peprotech, Rocky Hill, NJ, USA), and, in selected cultures, sodium acetate (1000 µM, S5636-250G, Sigma-Aldrich), sodium butyrate (500 µM, B5887-250 MG, Sigma-Aldrich), or sodium propionate (1000 µM, P5436-100G, Sigma-Aldrich) were added on day 0 and day 3. On day 5, the cells were harvested for phenotyping or further functional studies. The concentrations of SCFAs were chosen based on the immune cell viability (Supplementary Figure S1).

For the ChIP study, CD4^+^ cells generated from adult naïve non-Tregs were harvested on day 3, while those generated from CB were harvested on days 1 and 3 (Supplementary Figure S1).

For the flowcytometric analysis of Treg gene expression, iTreg cells were re-stimulated with anti-CD3 (OKT3, 4 µg/mL) for 3.5 h at 37 °C.

### 4.5. Suppression Assay

Allogenic responder cells for the suppression assay were enriched using a CD4^+^CD25^+^ Regulatory T Cell Isolation Kit (130-091-301, Miltenyi Biotec). Carboxyfluorescein succinimidyl ester (CFSE) was used to trace cell proliferation. Responder cells were washed (1300 rpm, 8 min) three times in RPMI-1640 in the absence of FCS at room temperature. T cells were then incubated (labeled) with 0.5 µM CFSE at 37 °C for 7 min in the dark. The addition of 200 μL of FCS could terminate the reaction. Afterwards, the cells were washed 3 times with RPMI-1640 with FCS. Non-CFSE labeled iTregs (or, where appropriate, CD25^high^ iTregs obtained from the whole population of adult iTregs using CD25 MicroBeads (130-091-301, Miltenyi Biotec) positive selection) and CFSE-labeled responder cells were co-cultured at a ratio between 1:1 and 1:16 and stimulated with Treg Suppression Inspector (cell: bead ratio of 1:1, 130-092-909, Miltenyi Biotec) in 96 U-bottom plates. The cells were cultured in medium for 3 days under standard culture conditions and subsequently harvested for flow cytometry. The percentage of suppression was determined as the difference in the proliferation between the negative control and the test sample, expressed as a percentage of the former.

### 4.6. Flow Cytometry

Flow cytometric analyses using BD FACSVerse (8-channel, Becton Dickinson) were conducted for all of the cultured cells, and a LIVE/DEAD™ Fixable Aqua Dead Cell Stain Kit (V500, L34966, Thermo Fisher Scientific) was used to determine the viability of all of the cultured cells prior to the fixation and permeabilization required for intracellular antibody staining. Surface and intracellular staining was performed according to the manufacturer’s instructions. For the intracellular staining of Foxp3 and CTLA-4, a True-Nuclear™ Transcription Factor Buffer Set (424401, BioLegend) was used. The antibodies used included CD4 (PerCP-Vio700, 130-093-504, Miltenyi Biotec), CD39 (APC, 130-109-455, Miltenyi Biotec), Foxp3 (Alexa Fluor 488, 320212, BioLegend), GITR (PE, 130-092-895, Miltenyi Biotec), ICOS (VioBlue, 130-100-737, Miltenyi Biotec), PD-1 (APC/Cy7, 329922, BioLegend), PD-L1 (PE/Cy7, 329718, BioLegend), and CTLA-4 (APC, 349908, BioLegend). The gating strategy is shown in (Figure 2).

The acquired sample (fcs) files were analyzed in FlowJo Version10 (Treestar, San Carlos, CA, USA). Consistent gating strategies were used for all samples in the same set of experiments. An isotype control was included to help with the gating. The mean fluorescence intensity (MFI) was calculated using the geometric mean.

### 4.7. Chromatin Immunoprecipitation–Quantitative Polymerase Chain Reaction (ChIP-qPCR)

Chromatin fixation, isolation, shearing, and immunoprecipitation, as well as DNA de-cross linking and purification, were conducted using the established and thoroughly validated in-house method described elsewhere [71,72,73]. For ChIP, a histone H4 pan-acetyl antibody (Anti-acetyl-histone H4 Antibody, 06-866; Merck, Darmstadt, Germany), targeting lysine residues 5 (K5), 8 (K8), 12 (K12), and 16 (K16) of the H4 histone, was used. Quantitative assessment of the H4 acetylation status was performed by the quantitative polymerase chain reaction (qPCR), as previously described [71,72,73], and the PCR primers used in the present study are given in Supplementary Table S1. In brief, the three-level strategy of PCR data normalization was applied. First, the percent enrichment to the input control was calculated for each target locus and a positive control gene encoding ribosomal protein L32 (*RPL32*), separately for the mock (IgG) and H4 antibody. Then, the locus-specific percent enrichment to the input control obtained for IgG was subtracted from the corresponding values for the H4 antibody. Finally, the calculated IgG-corrected percent enrichment was divided for each gene into that of *RPL32*, resulting in a relative enrichment value [71,72,74]. The intra- and inter-assay coefficients of variation calculated for the percent enrichment should not exceed 10% [71]. All samples were processed according to the same standardized protocol and analyzed blind and in a randomized order.

### 4.8. Statistical Analyses

Statistical analyses were performed in GraphPad Prism 9 (La Jolla, CA, USA). All of the data were not normally distributed due to the small sample size. Therefore, for comparisons among two or more groups, the Mann–Whitney test or Friedman test with Dunn’s multiple comparison test (*post hoc*) were used. For all analyses, a *p* value of less than 0.05 was deemed statistically significant.

## Data Availability

The data presented in this study are available on request from the corresponding author. The data are not publicly available due to privacy restrictions.

## Supplementary Materials

The following supporting information can be downloaded at: https://www.mdpi.com/article/10.3390/ijms23105740/s1.
