# Supplementary material for: Short-Chain Fatty Acids Augment Differentiation and Function of Human Induced Regulatory T Cells
Source: Int J Mol Sci. 2022 May 20;23(10):5740. doi: 10.3390/ijms23105740 (PMC9143307; doi:10.3390/ijms23105740)
Supplement: Supplementary file 1 [file ijms-23-05740-s001.zip › ijms-1709851-supplementary.pdf]

ONLINE SUPPLEMENTARY MATERIAL TO:

Short-Chain Fatty Acids Augment Differentiation and Function of Human Induced Regulatory T Cells

Mingjing HU*, **Bilal ALASHKAR ALHAMWE***, **Brigitte SANTNER-NANAN**, **Sarah MIETHE**, **Hani HARB**, **Harald RENZ**, **Daniel P. POTACZEK**[†], **Ralph K. NANAN**[†]

* Equal contribution
[†] Equal contribution

Supplementary Figure S1. General experimental layout of the study. Treg denotes regulatory T cell; CBMCs, cord blood mononuclear cells; PBMCs, peripheral blood mononuclear cells; αDC3/αCD28, anti-CD3/anti-CD28; TGF-β, transforming growth factor β; IL-2, interleukin-2; SCFAs, short-chain fatty acids; Acet, acetate; Buty, butyrate; Prop, propionate; ChIP, chromatin immunoprecipitation assay.

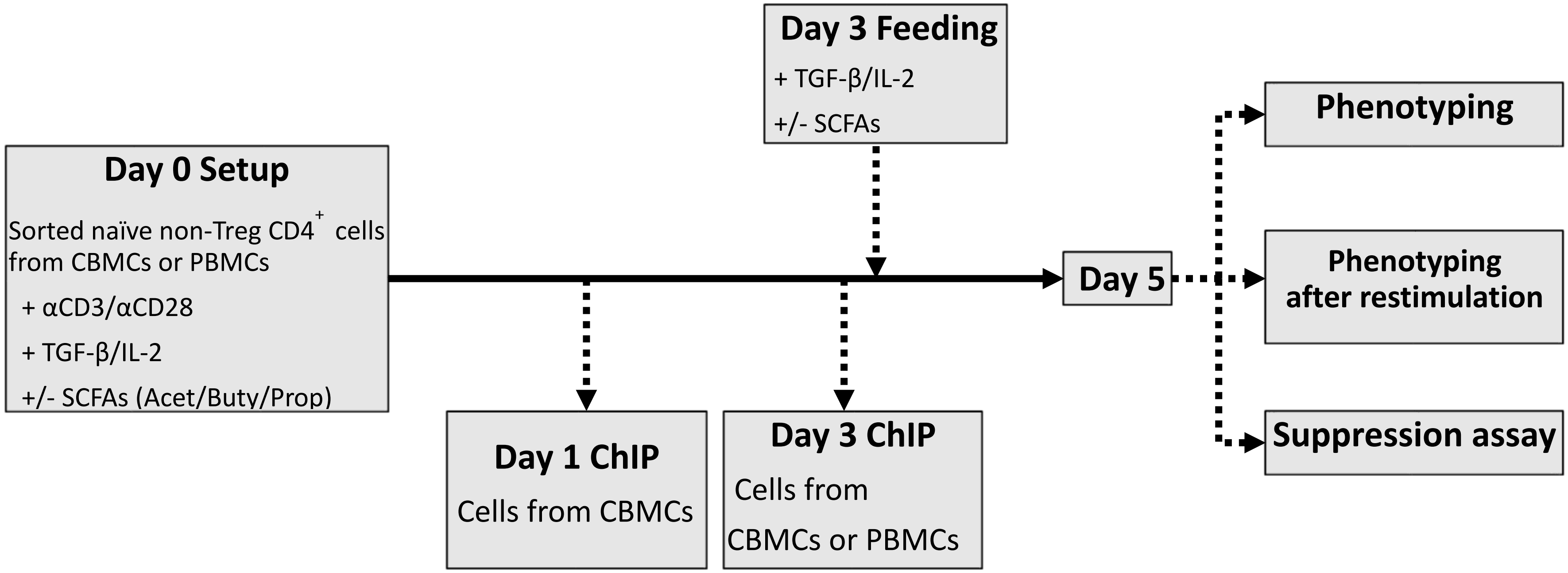

Supplementary Figure S2. Expression of phenotypic markers within Foxp3⁺ cells in both activated and non-activated cells generated from adult naïve CD4+ cells. Scattered dot plot with mean + standard error of the mean (SEM) showing the percentage of CD39⁺ cells, and mean fluorescence intensity (MFI) of GITR, ICOS, PD-1, PD-L1, and CTLA-4 gated on Foxp3⁺ cells of non-activated (Non-A) and activated (A) cells generated from adult naïve CD4+ cells under various conditions. All cells were cultured in the presence of transforming growth factor β and interleukin-2 and additionally with: control, acetate, butyrate, or propionate. Data summarize thirteen independent experiments. Wilcoxon matched-pairs rank test was used for statistical analysis. *$p < 0.05$, **$p < 0.01$, ***$p < 0.001$.

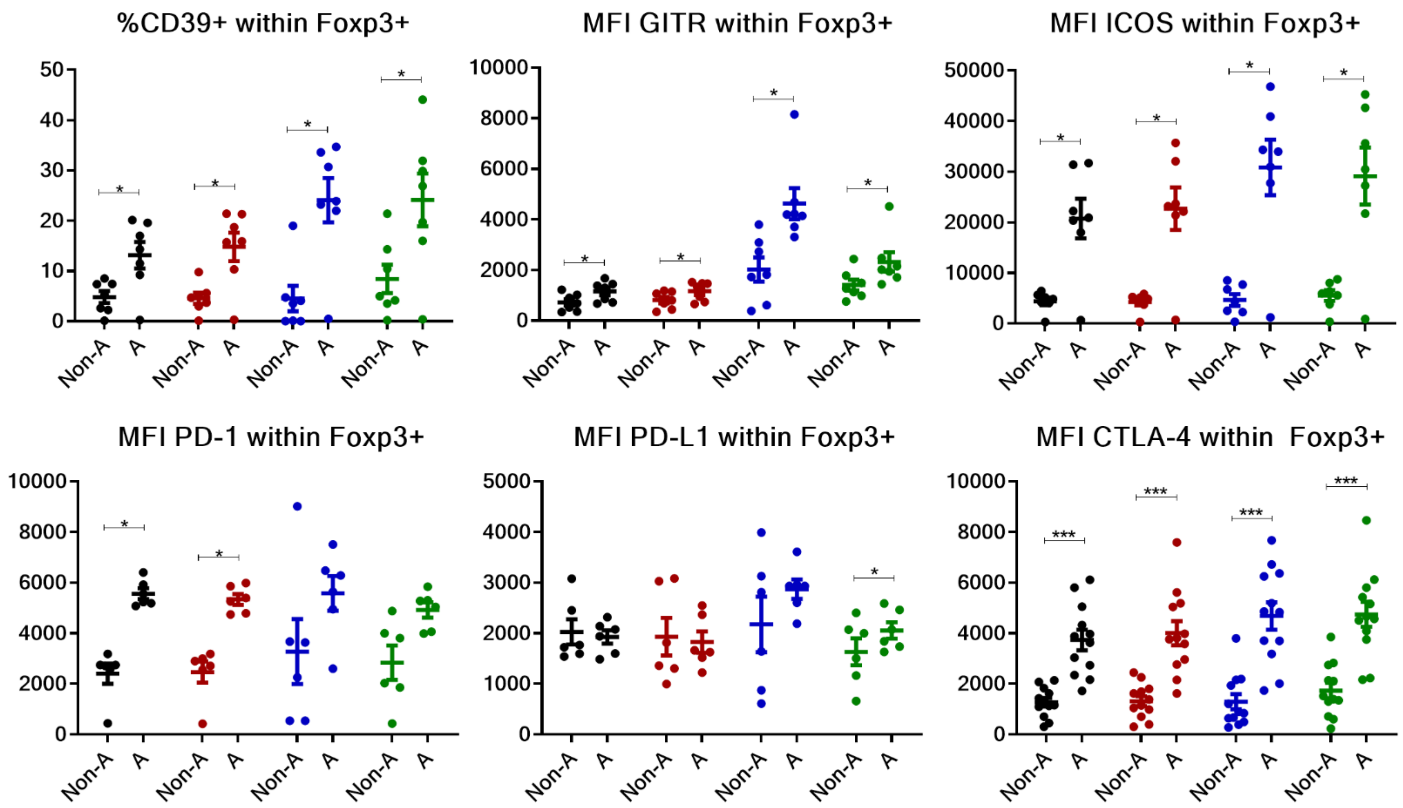

Supplementary Figure S3. Short chain fatty acids (SCFAs) potentiate the generation of human transforming growth factor β (TGF-β)-induced Tregs *in vitro*. Scattered dot plots showing mean + standard error of the mean (SEM) summarizing thirteen independent experiments for adult activated (Activated) and twelve independent experiments for cord (CB) bloods. Friedman test with Dunn's multiple comparisons (post hoc) was used to compare the expression of different markers between adult activated cells and cord bloods. *p < 0.05, **p < 0.01.

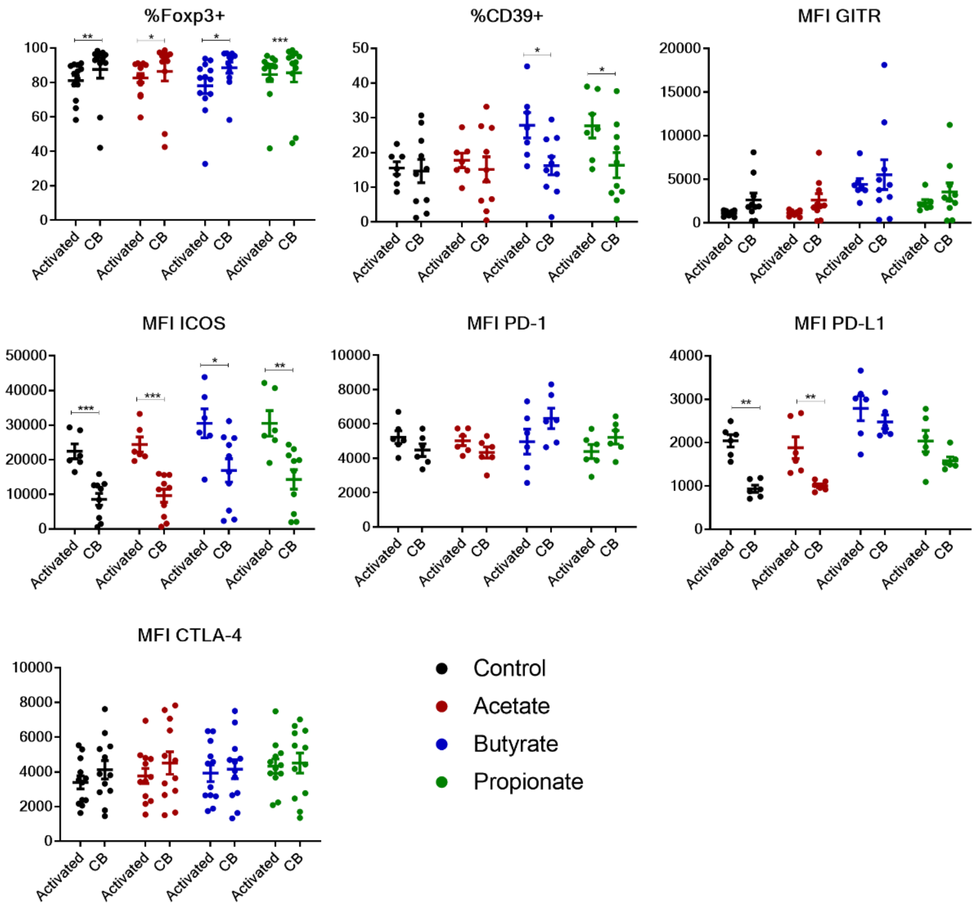

Supplementary Table S1. Primers used for quantitative assessment of H3 or H4 histone acetylation by PCR following chromatin immunoprecipitation (ChIP).

**Target**	**Forward primer**	**Reverse primer**
CD274 (PD-L1) molecule gene (CD274, *PDL1*) promoter	TCTTCCCGGTGAAAATCTCATT	TTCCTGACCTTCGGTGAAATC
Cytotoxic T-lymphocyte associated protein 4 (CTLA-4) gene (*CTLA4*, *CTLA-4*) promoter	TGCCTAGACAAATCCTGCCA	TCCTGGAGTACAAGGGTCCT
Ectonucleoside triphosphate diphosphohydrolase 1 (CD39) gene (*ENTPD1*, *CD39*) promoter	GTCCTGTCCCACATCCAGAA	TGACAGTGTGAGACCCTGTC
Forkhead box P3 (Foxp3) gene (*FOXP3*) promoter	TTCTTTCCCCAGAGACCCTC	AGGGCTCATGAGAAACCACA
FOXP3 conserved non-coding sequence (CNS) 3 (CNS-3)	CCTTTACTGTGGCACTGGG	GGATTTTCTTGGCCCTGCAA
Inducible T-cell costimulator (ICOS) gene (*ICOS*) promoter	TGATTCAGAGAAGTAGGGTGGT	TGCTGGAAAGGAAGTGGGTT
Programmed cell death 1 (PD-1) gene (*PDCD1*, *PD1*) promoter	AAGATCTGGAACTGTGGCCA	CTCAACCCCACTCCCATTCT
Ribosomal protein L32 (RPL32) gene (*RPL32*, *L23*; control gene)	GGAAGTGCTTGCCTTTTTCC	GGATTGCCACGGATTAACAC
TNF receptor superfamily member 18 (GITR) gene (*TNFRSF18*, *GITR*) promoter	GGCTCCATGGTTGAGGCTCT	GCGGCGTTTATAGCACCTTGT

All primers used for ChIP-PCR in this study were synthesized by Biospring GmbH (Frankfurt, Germany).

Supplementary Table S2. Sums of average histone 4 acetylation levels at seven Treg loci in human adult (n = 7; peripheral blood) or newborn (n = 6; cord blood) naïve $CD4^+$ non-Treg cells being differentiated toward iTreg phenotype in the presence or absence of short-chain fatty acids (SCFAs).

Donors	Harvesting day during 5-day differentiation	Control (no SCFA)	Acetate	Butyrate	Propionate
Adult	Day 3	4.318	5.139	9.330	10.393
Newborn	Day 1	6.973	5.582	10.918	8.184
	Day 3	2.811	3.376	4.186	6.278
